# FORGE: A Framework for Organizing Rewards in Gamified Environments

**DOI:** 10.1177/15554120241241555

**Published:** 2024-03-31

**Authors:** Rogerio de Leon Pereira, Olivier Tremblay-Savard

**Affiliations:** 1 Department of Computer Science, 8664 University of Manitoba, Winnipeg, Manitoba, Canada

**Keywords:** gamification, gamification framework, gamification for education, rewards, reward system, gamified environments

## Abstract

Rewards are prevalent game design elements in gamified environments. Rewards are helpful tools to keep players engaged and encouraged to perform tasks. However, implementing a reward system from scratch in every gamified environment, such as citizen science games or educational applications, can be time-consuming. This article presents FORGE, a Framework for Organizing Rewards in Gamified Environments. FORGE is a free and open-source framework built to be easy to install, easy to integrate, flexible and customizable. It was designed to be used in a variety of different environments and scenarios, such as games, training software and interactive websites. The FORGE framework comes with two sample applications, a simple game concept and an educational web application, to demonstrate its capabilities and serve as an entry point for new adopters.

## Introduction

In game design, rewards are given to players in exchange of putting in the effort to complete a task or overcome a certain challenge. The use of rewards is widespread in games and gamified environments (Goh et al., 2015; Siu & Riedl, 2016). They can be presented in different formats, such as achievements, badges, unlockable content, etc. Rewards are responsible for stimulating players. This stimulus can be linked to the users’ intrinsic motivations, as it provides recognition for overcoming an obstacle or the satisfaction of progressing in the game. It can also be connected to the users’ extrinsic motivations, where the players can compare their performance with their friends and other players through leaderboards and ranking systems.

Incorporating a reward system in a gamified environment requires an investment, either by purchasing a third-party solution or building it from scratch. While there are some free and open-source options, finding a solution that meets every need without any modification is hard. From this point of view, creating a highly customizable, free, and open-source reward system to suit a wide range of applications can be an excellent contribution to application designers, software developers, and teachers who resort to gamification to engage their users.

This article aims to achieve these objectives by presenting FORGE, a Framework for Organizing Rewards in Gamified Environments. FORGE was designed to be open-source and usable in a great variety of games, applications, and interactive websites. One of its goals was to facilitate the implementation of gamification through the use of rewards in many different contexts in addition to games, such as in citizen science, in training, and in the classroom.

Another goal of FORGE that guided its development is for it to be easy to install, easy to integrate, flexible, and customizable. The main technological requirement for the user is to be comfortable with cloning a repository, setting up a server and linking the server to the FORGE database. Although these steps could be a bit challenging for users with less experience, they are explained in the documentation accompanying FORGE, which follows industry-recommended standards for distributing this kind of technology.^1^ In general, we believe that users with an intermediate level of experience with game development, such as serious game/application developers, will benefit the most from using FORGE. Indeed, adopting a ready-made solution for intermediate-level users is much more practical since cloning a repository and running a server is much simpler than developing a customizable reward system from scratch. More experienced developers could also benefit from the time savings of using FORGE, allowing them to spend more time on more complex tasks.

FORGE has already been used in the development of GeSort,^2^ a citizen science game based on analyzing the evolution of genomes (Singh et al., 2017). It is also used here to develop a game (FORGE-game) and a website application (FORGE-app). Both were created to test and improve the framework and are described in more detail in the fourth section. Both FORGE-game and FORGE-app are open-source and available in the FORGE repository. They serve both as examples and starting points for those who want to learn how to utilize the framework. The strategy of developing applications to exemplify the use of a software framework is commonly employed in the literature to demonstrate its capabilities and serve as a tutorial for new users (Fischer et al., 2018; Truong, 2021).

FORGE is organized into components, each with a specific purpose, such as the FORGE-server responsible for communication, the FORGE-ui for creating and configuring rewards, and the FORGE-engine for validating rewards, among others. All are simple to install and configure. One of the benefits of adopting the FORGE framework is that it saves time in developing a new application. The time it would take to plan and develop a reward system from scratch is reduced to the few lines of code necessary for the integration of FORGE.

## Literature Review

Keeping students engaged is a challenge for any educator. The level of student engagement is higher when there is a balance between task difficulty and student skill, and also, the task should be exciting and relevant (Shernoff et al., 2014). According to the flow theory (Csikszentmihalyi, 2000), there are four states of mind: anxiety, apathy, boredom, and flow. When the task's difficulty is greater than the student's ability, it leads the student to a state of anxiety. If the task is too easy, the student feels bored. If the task has no relevance or sense, the student becomes apathetic.

Flow is the state between anxiety and boredom; it is the optimal state for learning. It is a mental state of total absorption in a challenging and enjoyable activity (Nakamura & Csikszentmihalyi, 2009). Flow theory is also considered in games, and some are designed to adjust the game's difficulty according to the players’ skills, keeping them in what is called the flow channel (Schell, 2019). Increasing engagement is also a challenge in crowdsourcing (Morschheuser et al., 2016), environments where various tasks are outsourced to be carried out by *the crowd*, whether in citizen-science projects such as Phylo (Kawrykow et al., 2012), or by crowdworkers such as in Amazon Mechanical Turk.^3^

It is not always possible to adapt the difficulty of the task to the user's ability, especially in environments where the skill level is not homogeneous. One alternative strategy is to adopt techniques that increase user engagement, one of which is gamification. This technique is not intended to reduce the gap between the difficulty of the task and the user's skill but to transform the activity into something more pleasurable and playful.

### Gamification

Gamification is a strategy to enhance systems and activities to improve users’ motivation, engagement (Hamari, 2013, 2017), learning (Hamari et al., 2016), and knowledge retention (Dincelli & Chengalur-Smith, 2020) among others. This is done by creating experiences similar to those experienced in video games (Hamari, 2019). This is usually conducted by applying game design elements in non-game contexts (Deterding et al., 2011). It has been shown that just framing an activity as a game, even without using actual gameplay mechanics, can increase the interest and enjoyment of participants (Lieberoth, 2015).

In gamified environments, user engagement can emerge through the pursuit of rewards when performing tasks or through competition. Reward types include points, progress, badges, achievements, levels, or providing some kind of virtual currency (Hamari & Eranti, 2011; Sailer et al., 2014). The advancement in the story by unlocking more game lore or even receiving access to educational content (de Leon Pereira et al., 2021) can also serve as motivational factors.

Competition can be stimulated by leaderboards or rankings, where individual achievements are visible and comparable among the users’ community. Leaderboards can serve as an achievable goal, where people feel encouraged to seek the first positions, even if they are not instructed to do so (Landers et al., 2017), which in the case of a school environment can lead to an increase in knowledge gain (Ortiz-Rojas et al., 2019). Nonetheless, using a leaderboard is not always advisable because, despite helping with engagement, it can negatively affect intrinsic motivation for some players who do not appreciate the added competition (Mekler et al., 2013; Xu et al., 2021).

### Gamification Elements

Although there exists many gamification elements and mechanisms (Reeves & Read, 2009), in this article, we are focusing on those related to reward systems which are described below.

#### Points

One of the main effects of points is to provide immediate feedback informing the user on how well they performed on a given task. Moreoever, it has been shown that displaying an achievable score (such as a top score) can increase player performance in a game (Matin et al., 2020). Points can also be distributed using scaling systems, as in the three-star system, where one star means that the activity was completed satisfactorily, and three stars indicate that the execution was done with mastery (Gaston & Cooper, 2017). This feedback loop is essential not just for learning but for the dynamics of motivation. The fact that points allow users to measure how much they are improving on a given task can also be a motivational factor (Dweck, 2006, 2013). Points can also represent progression, dedication, and commitment to completing tasks over time, such as experience points. Finally, points can be used as an in-game currency, where the players can spend them to acquire other rewards, such as cosmetic items or other assets (Zichermann & Cunningham, 2011).

#### Achievements, Badges, and Challenges

Achievements are normally used to reward players for completing a group of tasks or certain specific tasks that are more difficult or require more time and effort to complete. Badges most often serve the same role as achievements in games; however, badges visually represent the reward (Antin & Churchill, 2011; Werbach & Hunter, 2015). Badges and achievements also represent the player's performance over time or can be shared with community members who have earned the same reward. This element can also be used to encourage a user to perform certain actions, making the achievement more important or interesting than the tasks themselves (Wang & Sun, 2011). Challenges are a type of achievement that involves some time limit or time pressure. Challenges can be dynamically created to encourage users to participate in specific activities, for rewards that are not always available or present in the normal flow of the game. Challenges can also be designed to help a collaborative system, to guide users toward accomplishing tasks that are beneficial for the interactions between them (Tremblay-Savard et al., 2016).

#### Content

Content also serves as a form of reward. It can take the form of new levels, more character choices, difficulty levels, and other gameplay elements. Content can also be related to the story the user is experiencing. In some cases, the content may be supplementary information, whether or not it gives users advice or other advantages. The delivery of parts of a story as a reward between the completion of tasks serves as microdiversions, potentially contributing to increased performance and user retention (Dai et al., 2015). Giving sparse information after completing tasks can also stimulate curiosity, leading users to stay focused on the task to access more information, which also contributes to increased engagement (Law et al., 2016).

### Existing Tools and Frameworks for Gamification

The literature on gamification is vast (Mora et al., 2017) and it has been shown that in addition to increasing engagement, different gamification elements can be used to enhance learning outcomes (Kim et al., 2018), performance in a task (Matin et al., 2020), and collaboration (Tremblay-Savard et al., 2016). A starting point for a developer who wants to include gamification in their application is to find a tool that meets their needs.

One option is to purchase a paid tool. Bunchball^4^ is a gamification tool designed for the corporate environment to improve the performance and engagement of customers, partners, and employees. The tool receives data from user interactions and provides feedback to them. It offers real-time data-achievement feedback and recognition to keep its users on track and help them with course correction. Even if it is possible to adapt corporate solutions such as Bunchball for other gamified environments, high costs can impede their adoption. Even when a tool is more affordable, there is no way to predict how long it will be available. Badgeville, for example, is a gamification tool often seen in papers published in the first half of the 2010s. However, it was acquired in 2016 by CallidusCloud, which in 2018 was acquired by SAP,^5^ being currently only available as a package within the SAP platform.

There are also free options like Open Badges.^6^ Anyone can issue, display or host Open Badges through free and open specifications. However, Open Badges is not a general reward system but instead focuses on a single type of reward (badges). One of its advantages is that badges are verifiable, as they are digital certificates, but this goes far beyond what is necessary for most applications. Another free option is to adopt an open-source tool, such as UserInfuser.^7^ This framework supports the use of some gamification elements, such as badges, points, live notifications, and leaderboards. It also provides analytics to measure user participation. It is possible to adopt the tool as it is, or make some limited customizations to the gamification elements provided in the tool.

In addition to commercial and open-source options, several frameworks were created in the context of academic research, which are also available for free. One example is *GFramework*, a framework for gamifying web applications (Choi & Do, 2019). One of its advantages is that GFramework can generate game mechanics with some general information so that the programmer can develop gamified applications without the help of the gamification experts. However, the framework requires the use of its reward model called the noble reward model, which limits its customizability. Another example is a framework for gratifying software engineering activities (Sripada et al., 2016). Despite being robust and well-structured, this option is focused on a specific domain (software engineering), requiring additional effort to adapt it to other use cases. The problem with not having a general enough framework that can be easily customized and integrated into any application is that developers have to build their own specific systems all the time.

## Forge Components and Organization

Our framework was built on three pillars: (1) ease of installation, (2) ease of integration, and (3) flexibility and customizability. The aim is for it to be general and straightforward enough so that it is easy to adapt to as many applications and environments as possible. The framework user can create as many different types of rewards as needed, group them into categories and define how the system will release each of them. Rewards can be as varied as possible, such as points, badges, achievements, content, polls, quizzes, and others. Rather than building an entire reward system from scratch, the programming effort only focuses on integrating the framework into a new game or application.

In the context of FORGE, a reward is represented by an object containing a title, an image, a body, and a category. Only the reward title is mandatory; the other properties are optional, which makes the system more flexible and adaptable. The title is plain text, limited to 256 characters, and serves to identify the reward. The image can be uploaded on FORGE in JPG, PNG and GIF formats or a URL pointing to a figure on the internet can be entered. The body is text with no character limit that accepts basic HTML formatting (more details in the FORGE-ui section). The category serves to identify the type of reward, and the user can create as many categories as necessary.

To receive a reward, it is necessary to send a message to the server. In the context of FORGE, a message is an object containing a variable name and a variable value (more details in the FORGE-server section). The rewards are linked to targets. A target is an object that contains a variable name, a variable value, a logical or arithmetic operation, and a reward. The variable is a string that must start with a character and cannot contain spaces or special characters. The value is a number that can be positive, negative, integer, or fractional. The operation is a symbol that will be used to compare the variable and the value. The reward is the object that will be returned to the application that sent the message to the server if the condition established in the target is satisfied.

The first step for installing the FORGE framework is downloading the source code, which can be done directly from the *git repository*^8^ or using the *git clone* command on the command line interface. The second step is to use *npm* (node package manager) to download all the dependencies needed to run the FORGE framework components. The third and final step is to turn on the FORGE-server, and the framework is then ready to be used.

**Figure 1.**
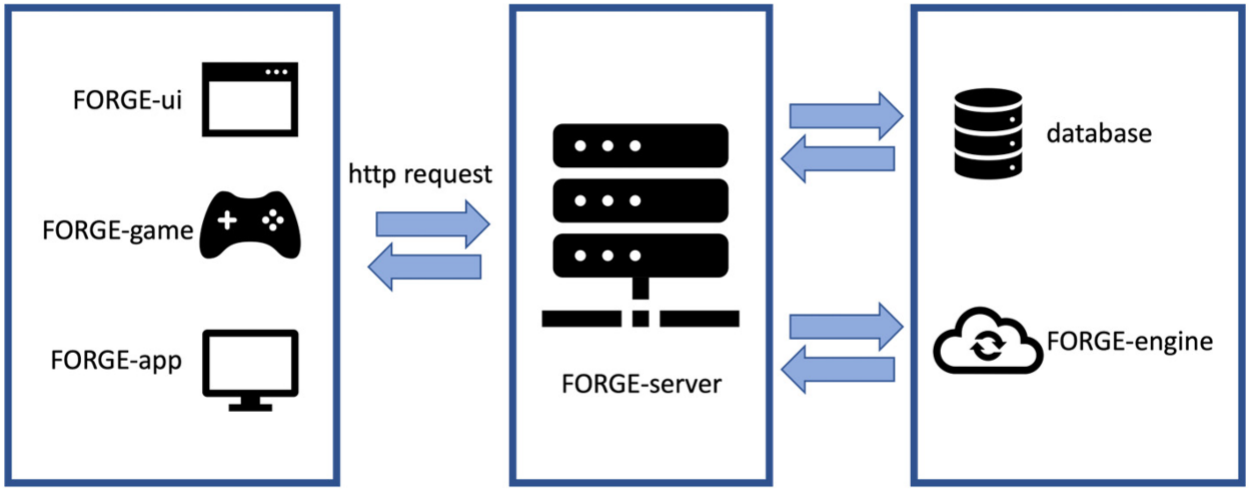
The FORGE components.

These steps and the list of each of the necessary commands are available in the documentation found in the *git repository*.^8^ The framework is organized into components as shown in Figure 1 and the languages used/supported by each component are presented in Table 1.

**Table 1.** FORGE characteristics.

FORGE components				
	Server	Engine	Database	UI
Language	JavaScript	JavaScript	NoSQL	JavaScript, JSX, HTML and CSS
Technology	Node.js, Express, Mongoose	Node.js	MongoDB	React
Platform	Unix, Linux, Windows, MacOS			

The integration process is simple. The application needs to send messages to the FORGE-server through an HTTP request, using one of the endpoints available in the API. This communication with the server can be done with a few lines of code. The FORGE framework has two example applications in its repository, a game made in Unity 3D and a website created in HTML and JavaScript. In addition to the documentation available, both applications (presented in the Proofs of Concept section) act as a starting point for learning how to use the FORGE framework in practice.

### FORGE-server

The FORGE-server component (Figure 1—center) handles all communication to and from the framework. It accepts HTTP requests containing an array of message objects. It sends those messages to the FORGE-engine for evaluation. The FORGE-engine will evaluate all messages received and return an array of rewards to the FORGE-server. The FORGE-server will connect to the database to retrieve the reward objects and send them back to the requester. An empty array is returned if no rewards are available based on the messages received.

The decision to prioritize array-based communication mediated by the FORGE-server rather than individual messages can mitigate situations when the server experiences temporary outages or when the application is offline. Adopting this approach, the application can accumulate messages during such periods and transmit them as a batch once the connection with the server is reestablished. This ensures that no messages are lost and improves overall communication reliability.

### FORGE-engine

The FORGE-engine (Figure 1—right) is the heart of the framework. It is responsible for evaluating messages and checking for available rewards. Recall that a *message* object is composed of a variable name and value. The *target* object has a variable name, a variable value, an operation, and a reward.

During the evaluation process, first the targets matching the variables received in the message are found. Suppose one or more targets satisfy this first condition. In that case, the variable value contained in the message is compared with the value in each of the matched targets, according to the operation registered in each target. If the result is true, the reward is added to an array, and the process is repeated for each message. The engine evaluates each message individually, and each evaluation may return none, one or more rewards. The reward array (which may be empty) is sent back to the FORGE-server at the end of the process.

### Database

**Figure 2.**
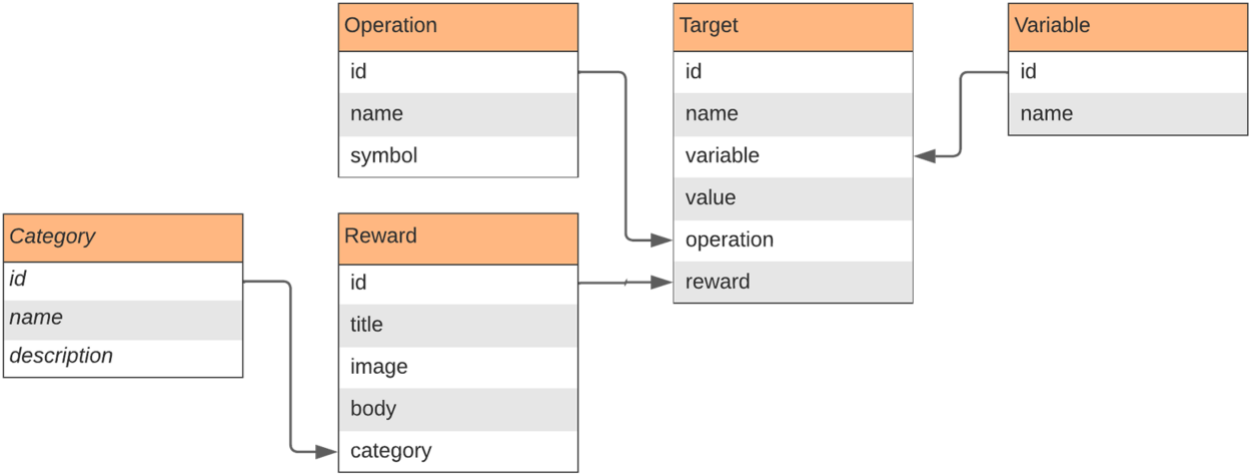
The FORGE database collections.

The database (Figure 1—right) is organized into collections (Figure 2). The main collection is that of rewards. It stores the title, image, body, and category. As the framework is flexible, it is up to the user to decide which reward categories are used in their gamification process. It could be badges, achievements, content, or anything that could describe a group of rewards. Another important collection is that of variables. There, the user registers the variables monitored in their game or application. The collection of operations contains a set of logical and arithmetic operators used to define the requirements for obtaining rewards. To link the pieces together, the user builds targets. A target selects which reward to receive, which variable to evaluate, which operation to use, and which value to achieve.

Some detailed examples are presented in the Proofs of Concept section.

### FORGE-ui

**Figure 3.**
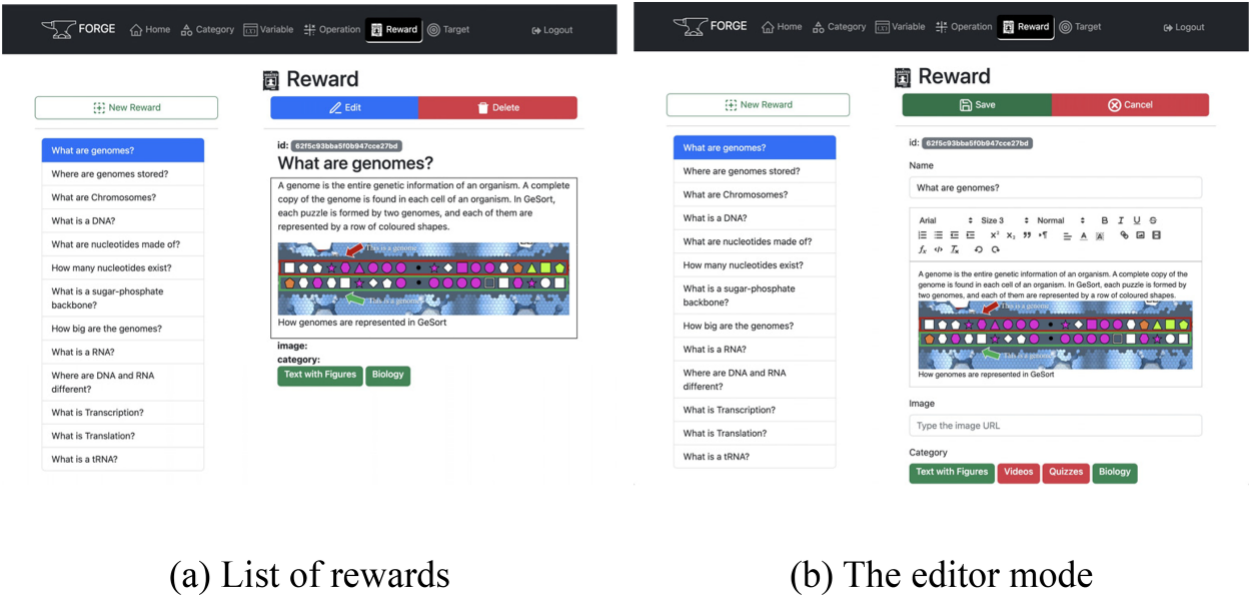
The FORGE-ui. The left side (a) shows the list of rewards with one reward selected. The *New Reward* button can be used to open the editor to add a new reward. The *Edit* button opens the selected reward in the editor for modification. The right side (b) shows one reward in the edition mode and the details of the *what you see is what you get* editor. This is an example of a reward that is educational content in GeSort, a citizen science game that makes use of FORGE (see the Citizen Science Game [GeSort] section).

The FORGE-ui component (Figure 1—left) serves as an interface through which the users can configure the framework by creating rewards and setting targets (Figure 3a). For the rewards body, FORGE-ui has a *what you see is what you get* editor. It contains the main HTML tags, available through a toolbar as shown in Figure 3b.

## Proofs of Concept

The framework is a reward system for games and gamified environments and was built to be a simple and easy-to-use tool. To demonstrate that, a game and an educational web application were created to test the framework in different realistic scenarios. This section introduces both and then describes the process of using the FORGE framework in GeSort, a citizen science game.

### FORGE-game (Example Game)

The objective of this first example is to demonstrate the versatility of the framework in the context of a game. The FORGE framework was used here to manage rewards such as *points* and *badges*. Besides the game-play logic, the game must prepare messages, send them to the framework, and apply changes to the game variables according to the answers received. Since the list of targets and rewards can always be queried from FORGE, it can be displayed inside the game and dynamically adjust to any updates made on the server.

The game was created using the Unity3D engine.^9^ This engine was chosen because it has a broad user base, has free and low-cost versions, a wide range of assets, good performance, and generates games for various platforms such as iOS, Android, several video game systems, desktop, and web browsers. Note that the FORGE framework can be used in any language capable of making HTTP requests, not just in games created with Unity3D.

**Figure 4.**
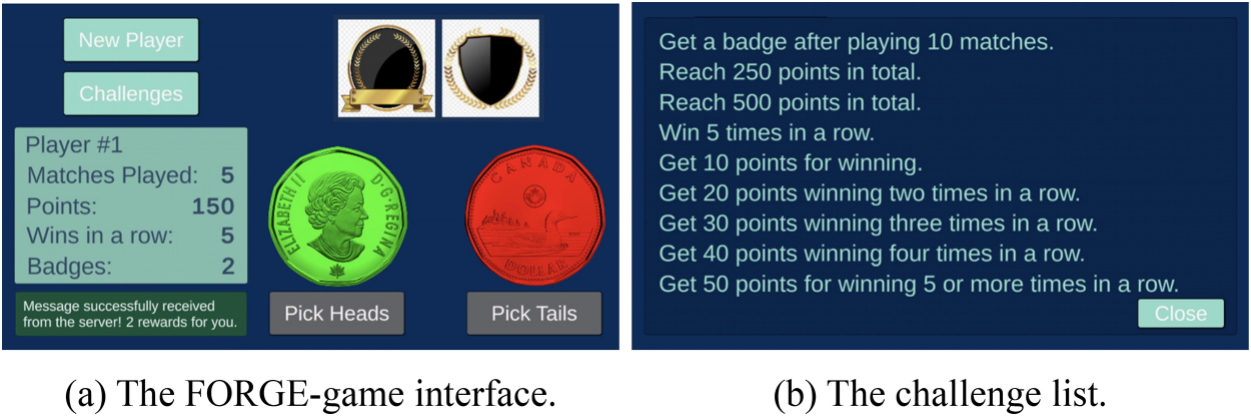
The FORGE-game. The left side (a) shows the game interface. The *New Player* button is on the upper left side to create a new player. The *Challenges* button is used to show the challenge list. Just below it, there is a box displaying the player information. In the upper right corner are the player's badges. At the bottom right are two coins, showing the *heads* and the *tails* faces. Clicking the *Pick Heads* button bets that the coin flip will result in heads and the *Pick Tails* bets that it will be tails. After a guess, the green coin shows the drawn face, and the red coin shows the opposite face. The right side (b) shows the challenge list that can be retrieved directly from the server, making it possible to add, remove, or change rewards without making changes to the game.

For ease of understanding, we made the example game as simple as possible (Figure 4). The player needs to guess the result of a coin toss and gets points for a correct guess. The game stores the player information into some variables, which will be sent to the framework after each play. The variables are: (a) *matches_played*, which means how many times the coin was flipped; (b) *points*, which are how many points the player has; and (c) *win_in_a_row*, which stores how many correct guesses have been made consecutively. The FORGE framework uses this information to determine rewards.

The FORGE-ui was used to set up the rewards. First, two categories were created, points and badges. Next, three variables were created using the exact names of the variables the game is prepared to send to the FORGE-server. For rewards, four badges and five different amounts of points were created.

The final step in FORGE-ui is to configure the targets. Recall that a target is an object that represents the necessary condition for a reward to be delivered. The process of creating a target requires setting the variable, expected value, operation, and reward. The list of targets used in this example game is shown in Table 2.

**Table 2.** List of available rewards and respective targets created in the FORGE framework for use in the FORGE-game.

Target	Variable	Operation	Value	Reward
Target01	matches_played	=	10	Badge01.png
Target02	points	*>*=	250	Badge02.png
Target03	points	*>*=	500	Badge03.png
Target04	win_in_a_row	=	5	Badge04.png
Target05	win_in_a_row	=	1	10 pts
Target06	win_in_a_row	=	2	20 pts
Target07	win_in_a_row	=	3	30 pts
Target08	win_in_a_row	=	4	40 pts
Target09	win_in_a_row	*>*=	5	50 pts

The game then only needs to verify the type of reward received. If it is a badge type, the game needs to download the image and display it in the interface. If it is a points type, the value is added to the player's total points. The game knows which variables to monitor and when to send them to the server. The server, in turn, only acts as an intermediary between the game and the engine. The engine doesn’t need to know how the game works; it just checks if there are any rewards available. In this way, the game and the reward system work independently. Thus, the application designer can create, delete or modify the rewards without making changes to the game, as long as the same variables are used.

### FORGE-app (Educational Web Application)

This website aims to show other possible framework uses, such as in an educational application designed for the classroom. The FORGE framework was used here to deliver educational content and quizzes to students, in a predetermined sequence. In this way, the teacher in this scenario can include or modify the order of educational materials whenever necessary. Besides the basic functionality such as user control and content display, the website must prepare messages, send them to the framework, and apply the necessary changes according to the answers received. The website was developed in HTML, CSS, and JavaScript. This technological combination is prevalent in Web 2.0 applications where pages are dynamic and respond to user interactions.

**Figure 5.**
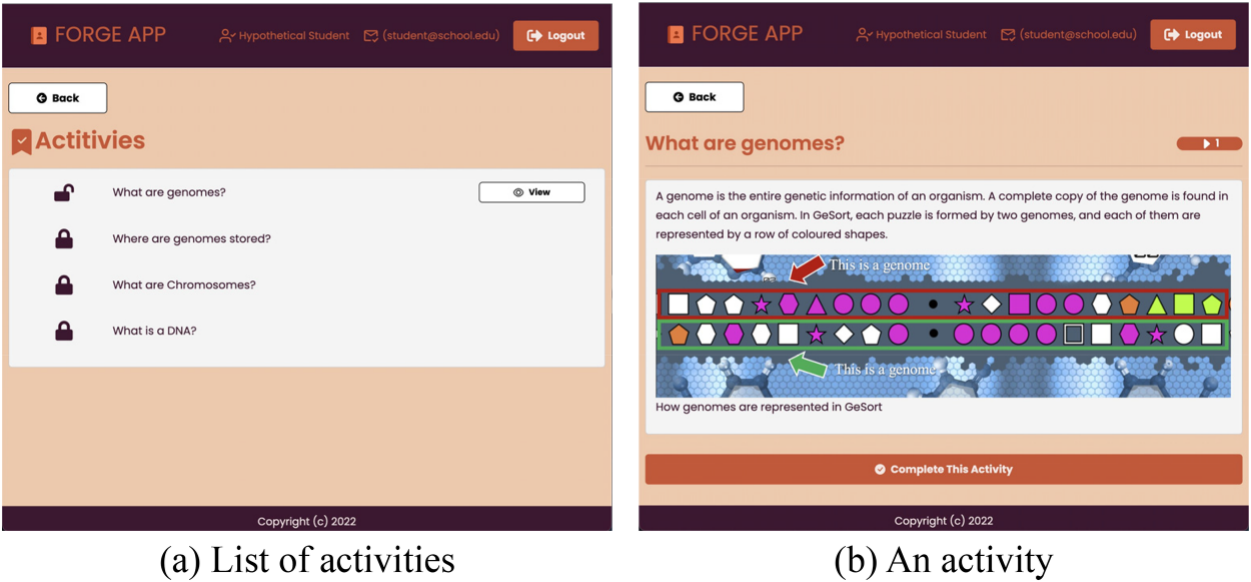
The FORGE-app. The left side (a) shows the list of activities presented to the student. There is a button labeled *View* to visualize the activity. In this figure, there is only one out of four activities that is currently available. The right side (b) shows one selected activity. At the bottom there is a button labeled *Complete This Activity* which the student should click after reading the content. Using this button will unlock the next activity (reward).

On the website (Figure 5), the student is presented with a list of activities upon logging into the system. The activity list is retrieved directly from the server. As a result, the website can dynamically adjust to changes made to the number and titles of rewards (activities) on FORGE by querying this list. Some of the activities are available while others are locked (Figure 5a). After clicking on an activity name, educational content (text, video, or quiz) is displayed (Figure 5b). After reading or watching the content, the student clicks on the *Complete This Activity* button to indicate that they are done. The website tracks an array containing the ids of the educational content already studied, and the variable *activity id* containing the id of the last viewed content.

Three reward categories were created using the FORGE-ui: texts, videos, and quizzes. Next, the variable named *activityID* was created, using the exact name of the variable that the application sends to the framework after each completed activity. The rewards created were three texts, three videos, and one quiz. The videos and the quiz are embedded in the body of the reward, but they are hosted on YouTube (videos) and Google Forms (quiz).

The last step to complete in the FORGE-ui is to configure the targets so that the student is rewarded with new educational content after having studied the available content. After going through all the materials, the student can work on the quiz. This way, the teacher can guide the students through a predetermined lesson/exercise. Creating targets requires to set the variable, the value, the operation, and the reward. The list of targets used here is shown in Table 3.

**Table 3.** List of available rewards and respective targets created in the FORGE framework for use in the FORGE-app.

Target	Variable	Operation	Value	Reward
Target01	activityID	=	null	Text01
Target02	activityID	=	Text01	Video01
Target03	activityID	=	Video01	Text02
Target04	activityID	=	Text02	Video02
Target05	activityID	=	Video02	Text03
Target06	activityID	=	Text03	Video03
Target07	activityID	=	Video03	Quiz01

### Citizen Science Game (GeSort)

**Figure 6.**
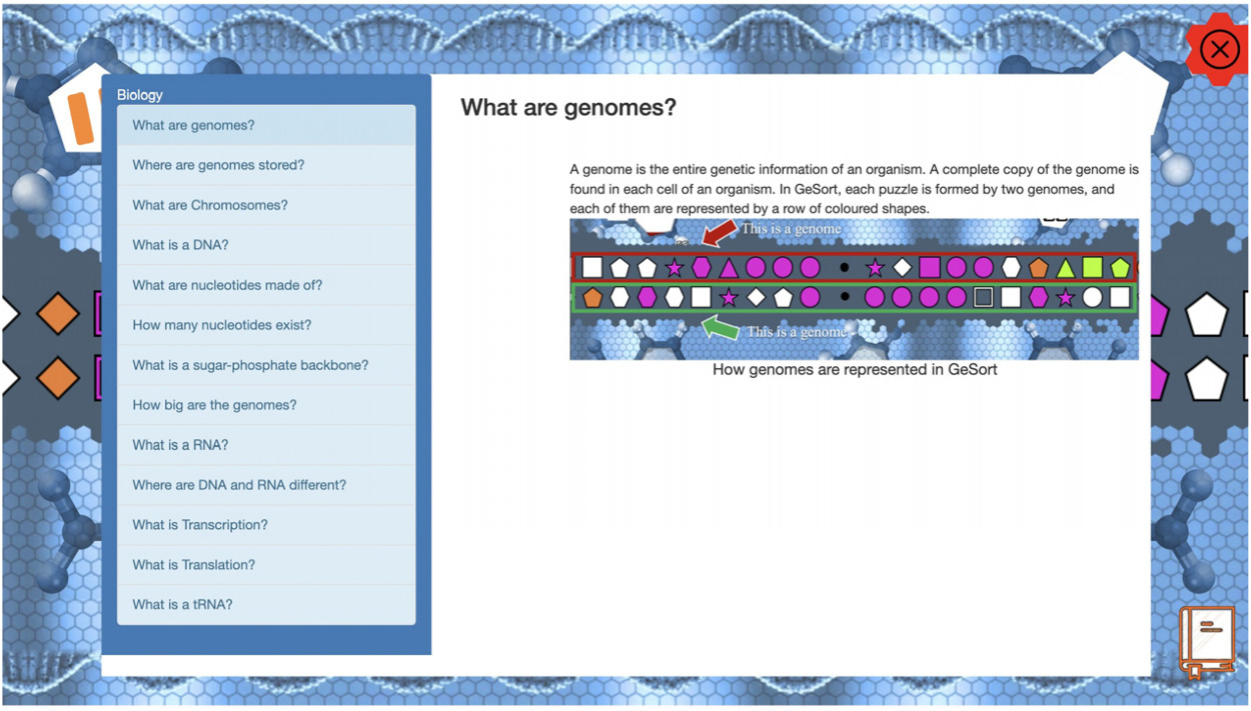
The GeSort game with the embedded educational content.

The framework was also used in the development of GeSort (Figure 6), a citizen science game that analyzes the evolution of genomes (Singh et al., 2017). From a game design perspective, GeSort is a puzzle matching game. The objective of the game is to transform a row of colored shapes into another one, using as few moves as possible. The game's design is based on a problem in comparative genomics, where the objective is to find the shortest evolutionary path between two genomes. This problem is known as the genome sorting problem, and in the game, the rows represent genomes, and the colored shapes represent genes.

In a recent study, the GeSort researchers were interested in evaluating the effects of including educational content in a citizen science game. The study was designed to have the participants randomly split into three groups, in which the participants in one group should receive gradual access to the educational content as they progress in the game (de Leon Pereira et al., 2021). FORGE was used in this context to gradually deliver the educational content as a reward for completing multiples of three consecutive puzzles.

Through the FORGE-ui, the authors created six rewards, corresponding to six educational content topics. The targets were defined by considering two factors: the group to which the participant was assigned and how many levels were played at the time of the reward's verification. Thus, one target was set for every multiple of three levels completed (up to 18) for a specific group of participants who had gradual access.

## Discussion

In this article, we presented FORGE, a framework for organizing rewards in gamified environments. The FORGE framework was built to be easy to install, easy to integrate, flexible, and customizable. The installation process requires only downloading (or cloning) the source code and a few command lines. We made the experience of installing the FORGE framework similar to that of installing traditional software. The integration process relies on setting up a server and including a bit of code in the application to send messages to the framework and handle the responses received. This is done with a few lines of code (about 5–15 in our examples), and the applications presented in the FORGE-game (Example Game) and the FORGE-app (Educational Web Application) sections can be used as a reference, given that their source code is available together with the source code of the FORGE framework.

Flexibility and customizability are the main strengths of the FORGE framework. The flexibility lies in the possibility of creating any type of reward. The object that represents a reward has a title, an image and a body composed of HTML tags. This ensures that the FORGE framework can be used for simple tasks, such as giving points or badges for completing a task, and any other more complex content that uses texts, videos, quizzes, surveys, or any other third-party feature that can be embedded into an HTML page (e.g., Google Forms, Microsoft Forms, etc.). Customization takes place through the definition of targets. The target, in the context of the FORGE framework, is an object that contains the rules that define how and when a reward should be delivered. This allows a high degree of customization, linking the different types of rewards to various events according to the end user's interaction with the application. The FORGE framework can be used in any application capable of making HTTP requests: it is not restricted to games made in Unity3D or websites made in HTML and JavaScript.

Another critical point is the independence between the application and the FORGE framework. The application does not necessarily need to know what the rewards are and what conditions are required for each of them. The application only needs to send messages to the FORGE-server and handle the data that is sent back from it. On the other hand, the FORGE framework does not need to know how the application works; it just gets the messages, processes them and sends back the response. In other words, the developer of an application can simply choose which variables should be sent to FORGE, and at which frequency (e.g., after completing a level, interacting with a page, or submitting an answer to a question). On the FORGE side, any reward can be created based on the values of these variables that are received regularly from the application. The application can then set up a generic protocol to respond to the list of rewards received (e.g., showing a dialog window of the newly obtained rewards, displaying a complete list of badges obtained, or displaying an icon to indicate that new content has been unlocked).

This independence between FORGE and the application is an important feature given the way applications are distributed nowadays. On the main distribution platforms such as STEAM Games, Apple Store, and Google Play, applications must be verified and approved before publishing, and this can create significant delays (days or weeks). For each update, the new version needs to be sent to the virtual store and go through another evaluation process before being available to the user. One can easily imagine a scenario where the rewards list needs to be frequently modified, for example to add new challenges to keep users engaged, or to create temporary challenges according to seasons or festive events. If a new version needs to be updated on the distribution platform for each occasion, a considerable amount of time will be spent in the validation process. This is one of the points where FORGE as a reward framework can help developers. The decoupling of the reward system and the application means that the rewards can change without modifying the original application. Of course, this is only valid if the new rewards are planned to consider the variables already being sent to the framework; otherwise, adjustments must be made to the application. Note that the rewards list can always be queried from FORGE so that applications can dynamically adjust to changes made on FORGE and display the list of currently available content or challenges.

On a different note, it is still unclear how much gamification is needed in different contexts to achieve a higher level of engagement from the user base. While adding a certain level of gamification usually helps, studies have shown that some gamification elements can sometimes negatively affect motivation and engagement (Alsawaier, 2018). In this context, the FORGE framework shows its potential. In the case of an application with too little gamification, it is simple to use the FORGE-ui to create new rewards and their respective targets. Conversely, if the gamification level is excessive and distracting, removing rewards is equally straightforward. All of this can be constantly fined-tuned without having to change a line of application code or go through the distribution platform approval process again.

## Limitations

We built two example applications to make the process of learning how to use the FORGE framework simple. This is a practice observed in the presentation of other frameworks, as in the case of iCub-HRI (Fischer et al., 2018) and IoTCloudSamples (Truong, 2021). FORGE was also deployed in one full-sized gamified project (GeSort) in the context of bioinformatics. Even with our best efforts to realize comprehensive testing, some potential situations and special cases might not have been covered. It would be interesting to see FORGE tested in the context of other games (citizen science or not), classroom settings, and training scenarios in all kinds of different domains, areas, and disciplines. We believe that continuous interactions with the community will be fundamental for the FORGE framework to reach its full potential.

## Conclusion and Future Work

The FORGE framework has the potential to open doors for gamification, given its ease of integration, allowing to save time and resources for developers who cannot afford to create a reward system from scratch. Its flexibility and customizability make the FORGE framework a tool that can be adapted to various situations, such as teaching, training, crowdsourcing, and citizen science scenarios. Even for applications that go through the approval process on digital distribution platforms, the FORGE framework allows new rewards to be included or modified without altering the original application.

In terms of possible technical improvements, the FORGE framework does not currently support targets with what we call *composite conditions*: when a target has more than one operation or more than one variable. Alternatively, it is currently possible to overcome this limitation by sending the variables separately and combining the results received from the server. In a future version of the FORGE framework, we will add this feature to ensure that all the evaluation work is carried out by the FORGE-engine.

Another avenue for future work is related to the interaction with other products, as in the case of third-party polling platforms (e.g., Google Forms). In the application presented in the FORGE-app (Educational Web Application) section, one of the rewards was the access to a quiz. This quiz was created using Google Forms, and the user can access it directly within the application. However, the FORGE framework cannot access the quiz results and use this information to release subsequent rewards. Imagine that the teacher defines that the student who gets a perfect score on the quiz should receive a badge. In this case, the teacher must access the Google Form, check the student's grade and then grant the badge. One option we see in the future is to use the APIs of popular polling platforms and have the FORGE framework directly query the forms and use the answers to evaluate other rewards. Another possibility is to have an option to easily integrate FORGE into virtual learning environments (VLEs) such as Moodle, as has been done with other gamification frameworks (Altaie & Jawawi, 2021).

## Notes

1.
https://docs.npmjs.com/packages-and-modules/contributing-packages-to-the-registry

2.
https://gesort.cs.umanitoba.ca/

3.
https://www.mturk.com/

4.
https://www.biworldwide.com/gamification/bunchball-nitro/

5.
https://www.sap.com/acquired-brands/what-is-calliduscloud.html/

6.
https://openbadges.org/

7.
https://code.google.com/archive/p/userinfuser/

8.
https://github.com/UofM-BioinformaticsLab/forge

9.
https://unity.com/
